# Long-Term Results of Radiofrequency Energy Delivery for the Treatment of GERD: Results of a Prospective 48-Month Study

**DOI:** 10.1155/2011/507157

**Published:** 2011-10-24

**Authors:** Luca Dughera, Monica Navino, Paola Cassolino, Mariella De Cento, Luca Cacciotella, Fabio Cisarò, Michele Chiaverina

**Affiliations:** ^1^Digestive Motility and Endoscopy Unit, Department of Medicine, ASO San Giovanni Battista Hospital, Via Genova 3, 10121 Turin, Italy; ^2^Emergency Surgery Unit, Department of Emergency, ASO San Giovanni Battista Hospital, 10121 Turin, Italy; ^3^Anesthesiology Unit, Department of Surgery, ASO San Giovanni Battista Hospital, 10121 Turin, Italy

## Abstract

Since 2000, radiofrequency (RF) energy treatment has been increasingly offered as an alternative option to invasive surgical procedures for selected patients with gastroesophageal reflux disease (GERD). Out of 69 patients treated since June 2002 to December 2007 with the Stretta procedure, 56 of them reached by the end of 2010 a 48-month followup. RF treatment significantly improved heartburn scores, GERD-specific quality of life scores, and general quality of life scores at 24 and 48 months in 52 out of 56 patients (92,8%). At each control time both mean heartburn and GERD HRQL scores decreased (*P* = 0.001 and *P* = 0.003, resp.) and both mental SF-36 and physical SF-36 ameliorated (*P* = 0.001 and 0.05, resp.). At 48 months, 41 out of 56 patients (72,3%) were completely off PPIs. Morbidity was minimal, with only one relevant but transient complication. According to other literature data, this study shows that RF delivery to LES is safe and durably improves symptoms and quality of life in well-selected GERD patients.

## 1. Introduction

GERD is a chronic condition involving spontaneous and involuntary reflux of stomach contents into the oesophagus most frequently caused by the incompetence of the lower oesophageal sphincter (LES). 

The prevalence of GERD in western countries ranges between 10 and 20 per cent [1]; treatment depends upon symptom severity and individual patient characteristics and often requires long-term medical therapy or laparoscopic surgery [2]. Despite that, about 20% of patients will have breakthrough heartburn and regurgitation and long-term use of medications, which provides a significant and life-lasting economic expense.

Since the main goal of treating GERD would be the achievement by the patient of a sustained better quality of life and neither medical nor surgical therapy meet the idealized criteria of being simple, effective, risk-free and nonexpensive, a wide number of endoscopic procedures to create an antireflux effective barrier, thus obviating the cost of long-term PPIs treatment and potential risks of laparoscopic surgery, have been recently described [3, 4].

In April 2000, the FDA approved the Stretta system (Curon Medical, Fremont CA, USA) for use as an endoscopic treatment for patients with GERD [5] that was increasingly offered as first-line therapy before more invasive surgical procedures for selected GERD patients, with clinical data supporting its efficacy, safety, and patient satisfaction [6–10].

The device imparts RF ablation to the dysfunctional lower oesophageal sphincter via the endoscopic balloon-mounted needles. Energy is applied at six to eight levels circumferentially around the oesophageal junction. Two main goals are achieved: first, scarring of the distal oesophageal muscular wall improves the reflux barrier of the lower oesophageal sphincter; second, reduced transient lower oesophageal sphincter relaxations occur due to ablation or demodulation of vagal afferent fibres in the vicinity.

This prospective study reports our experience using the Stretta procedure in strictly selected patients suffering of GERD who have been followed up for 4 years.

## 2. Materials and Methods

From June 2002 to December 2007, we have selected and treated a total of 69 patients with the Stretta procedure and 56 of them reached by the end of 2010 a 48-month followup. The device was not available on the market from late 2006 through the end of 2010, when the study protocol has been restarted.

Baseline characteristics of the patients are outlined in Table 1. 

Most patients had breakthrough symptoms while on pharmacologic treatment or were not willing to take drugs for a lifetime and/or refused laparoscopic antireflux surgery. 

Participants met the following criteria: (1) heartburn or acid regurgitation quite well responsive to daily medication with proton pump inhibitors (PPIs); (2) age >18 years; (3) 24-hour pH study (off medication) showing abnormal oesophageal acid exposure (≥4%) and a De Meester score of more than 14.7; (4) oesophageal manometry showing both normal peristalsis and sphincter relaxation and LES pressure below 10 mmHg and more than 5 mmHg; (5) at upper endoscopy (EGD) on medications, no evidence or low-grade oesophagitis (Los Angeles grade A-B) [11], no hiatal hernia or not longer than 2 cm, and no Barrett's oesophagus; finally, (6) coagulation disorders, previous oesophagogastric surgery, relevant cardiovascular, and metabolic diseases, cancer, psychiatric disorders, or nutritional behaviour disturbs such as anorexia and bulimia were excluded. Patients showing at manometry significant ineffective oesophageal motility (IEM) associated with GERD were also excluded. 

The institutional review board approved the protocol and all patients provided written informed consent.

The procedure was performed using the technique first described by Triadafilopoulos [12]; during a deeply sedated EGD, the operator confirms the eligibility criteria and measures the position of the squamo-columnar junction (used as the approximate location for the gastroesophageal junction); then the endoscope is withdrawn and the RF delivery catheter was introduced orally over a guide wire. The Stretta catheter consists of an inflatable and flexible balloon-basket with four electrode needle sheaths. The operator inflates the balloon 2 cm proximal to the squamo-columnar junction, deploys the electrode needles (22-gauge, 5.5-mm length), and delivers RF energy for 90 seconds; since 2005 the RF delivery protocol was changed and a highest RF energy was subsequently delivered only for 60 seconds. The needles are then withdrawn, the balloon deflated, and the catheter rotated 45°. This process is serially repeated every 0.5 cm, covering an area 2 cm above and 1.5 cm below the squamo-columnar junction plus six sets below the cardias, for a total of 22 sets of needle deployments.

All procedures were performed on an outpatient day-hospital basis; for deep sedation we employed propofol (100–300 mg i.v.) and remifentanil (0.5–1 mg/kg/h i.v.), with continue cardiorespiratory monitoring by an anesthetist well trained in assistance to the endoscopic procedures. Recovery mean time after procedure was 4–6 hours, and all patients were discharged from the hospital within the day.

The median procedure time (from starting the initial endoscopy to the catheter removal) was 50 minutes (45–70 min). EGD was performed immediately posttreatment to evaluate the RF-induced lesion placement. All patients continued their current PPIs regimen for 30 days and then discontinued all antacid medications. For symptom recurrence, a standard step-up protocol was used, starting with antacids followed by H2-receptor antagonists and PPIs until symptom relief was achieved.

The primary outcomes of the study were heartburn (using a 6-point Likert scale ranging from no symptoms to incapacitating symptoms), GERD health-related quality of life (HRQL), using a 6-point Likert scale for multiple different symptoms, each ranging from no symptoms to incapacitating symptoms [13], and general quality of life, using the medical outcomes 36-item Short-Form Health Survey (SF-36) [14].

GERD HRQL improvement was evaluated as a continuous variable and as a dichotomous variable (responder versus nonresponder). Complete 24 and 48 months clinical data were available for all patients. A response was a >50% improvement compared with baseline values, as previously described [15].

Secondary outcomes included medication use (using a questionnaire) and LES pressure at oesophageal manometry. Adverse events were evaluated by using patient questionnaires and reported on a dedicated report form.

For statistical analysis, normality was assessed graphically and with the Shapiro-Wilkes test. For normally distributed variables, we reported mean values and performed comparisons with unpaired and paired *t*-tests, as appropriate. For variables without normal distributions, we reported median values and performed comparisons with the Mann-Whitney two-sample statistic or the Wilcoxon-matched pairs signed rank test, as appropriate. Analyses of dichotomous data (e.g., medication use) used the chi-square statistic.

## 3. Results

Thirteen patients out of the 69 treated by RF delivery at December 2007 were excluded from the data analysis: 9 patients did not reach by the end of 2010 the 48-month follow-up visit, 2 patients were lost at followup, and in 2 patients the Stretta procedure was completely ineffective within 6 weeks from the RF delivery and underwent laparoscopic antireflux surgery without any technical difficulty.

Fifty-six patients reached the 48-month follow-up and accepted to undergo oesophageal manometry at 24 and 48 months and EGD at 48 months. 

**Figure 1:**
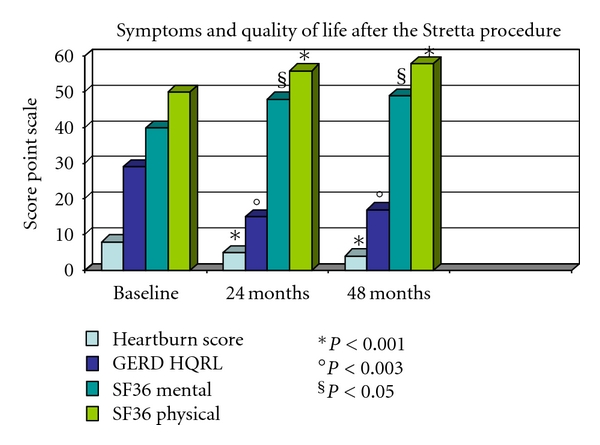
Means report 95% confidence intervals, medians report 25th and 75th percentile ranges, and proportions report percentiles. Heartburn score used a 6-point Likert scale: 0, no symptoms; 1, symptoms noticeable but not bothersome; 2, symptoms noticeable and bothersome, but not every day; 3, symptoms bothersome every day; 4, symptoms affect daily life; 5, symptoms incapacitating (unable to perform daily activities). For symptom scores, the statistical tests compared the mean/median differences in absolute change from baseline values. Heartburn and heartburn-related quality of life (HRQL) scores when off antisecretory medications (higher scores for worse symptoms). SF-36 physical score (higher scores for better function).

**Figure 2:**
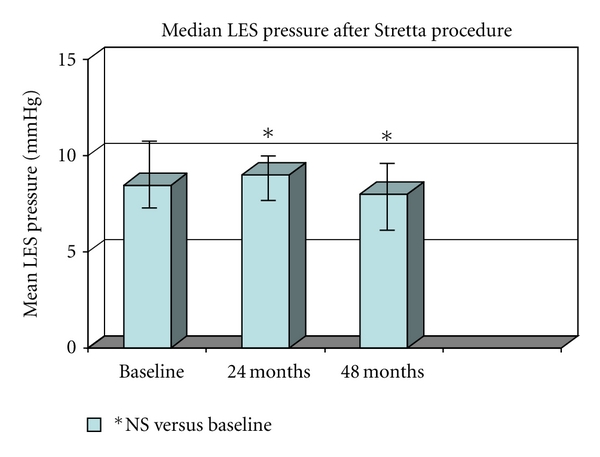
Mean Less pressure was evaluated by oesophageal manometry; median basal pressure was 8.44 mmHg (7.2–11.7), at 24 months was 9.5 (7.8–10.2) and at 48 months was 9.1 (6.9–9.2). No significant differences from baseline values were found.

RF treatment significantly improved heartburn scores, GERD-specific quality of life scores, and general quality of life scores at 24 and 48 months in 52 out of 56 patients (92,8%). At each control time, both mean heartburn and GERD HRQL scores decreased (*P* = 0.001 and *P* = 0.003, resp.) and both mental SF-36 and physical SF-36 ameliorated (*P* = 0.001 and 0.05, resp.). (Figure 1). Whereas in our cohort of patients the median LES pressure was previously described to increase at 12 months [15, 16], only an overall not significant effect from baseline and posttreatment values was finally observed at 24 and 48 months (Figure 2). 

We did not find any difference in the long-term clinical outcome between patients who underwent to RF delivery for 90 seconds and 60 seconds, but these second ones received a significantly lower dose of anesthesia and had a shorter recovery time.

At the endoscopic followup at 48 months, none of the treated patients showed oesophagitis or a picture of Barrett's oesophagus.

**Figure 3:**
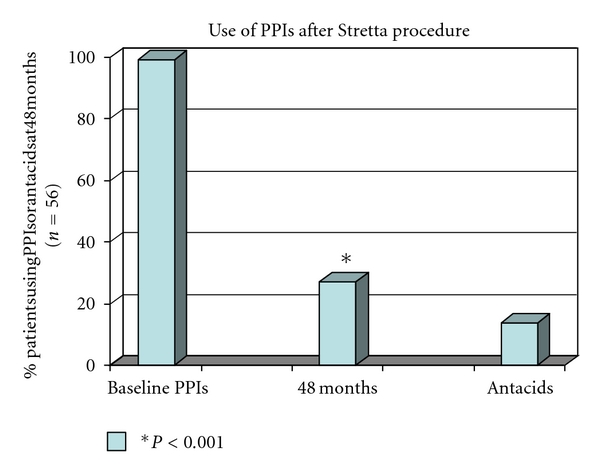
The Stretta procedure reduces significantly the use of antireflux medication, at 48 months, 41 out of 56 patients (72,3%) were completely off PPIs (including OTC-PPIs and anti-H2 antagonists); some patients (14%) were still using only occasionally oral antacids, none of them on a weekly basis.

At 48 months, 41 out of 56 patients (72,3%) were completely off PPIs (including OTC-PPIs and anti-H2 antagonists); some patients (14%) were still using only occasionally oral antacids, none of them on a weekly basis (Figure 3). 

There were no post procedure perforations, mucosal lacerations, bleeding episodes requiring transfusion, or deaths (Table 2). Minor complications were temporary postprocedure chest discomfort, requiring only oral analgesics, mild fever, transient nausea, and vomiting and transient dysphagia. The only major adverse event was a prolonged transient gastric paresis in a 52-year-old male patient. The patient had to be hospitalized for 3 weeks and treated with prokinetics, prostigmine, and enteral nutrition. The problem was completely resolved within approximately 8 weeks.

## 4. Discussion

This study evaluated multiple outcomes, including symptom scores, GERD-specific quality of life, and general measures of quality of life, in 56 consecutive patients submitted to the Stretta procedure, with a followup of 48 months and without dropouts. It also directly evaluated oesophageal sphincter pressure before and after treatment.

Like all other published studies on the RF treatment for GERD, the current study excluded patients with large hiatal hernias, severe grade C-D erosive oesophagitis despite medical treatment, Barrett oesophagus, or primarily extraesophageal manifestations of GERD (e.g., asthma); such patients should not be treated outside of especially designed research protocols.

Human and animal studies suggest that RF energy may ameliorate GERD reducing the frequency of transient LES relaxations associated with reflux episodes, increasing the intragastric pressure needed to induce reflux and improving the gastric emptying [17–19]. Another potential mechanism for ameliorating GERD symptoms is the decreased visceral sensitivity, as suggested by the observation that the time needed to report symptoms during oesophageal acid perfusion was significantly longer than 6 months after the Stretta procedure [20]. RF energy delivery may influence several of these mechanisms as well as the compliance of the gastroesophageal junction, increasing the resistance of the LES to a distending pressure and decreasing the volume of acidic refluxate while the LES relaxes.

In this study, we found that RF energy delivery to the gastroesophageal junction significantly and durably reduced GERD symptoms, improved GERD HRQL and general quality of life, and substantially reduced the use of PPIs. Our clinical data meet with most of the literature results regarding long-term followup [10, 21–23]. 

Limitations of these reports include the use of a single community practice and a nonrandomized or comparative nature of the studies, but a single-center study is not by itself of minor significance. As such, it sustains the importance that these procedures would be still restricted to study protocols with well-defined patient selection and to well-experienced and specially trained endoscopist [24]. At our faculty, all the RF delivery procedures were performed by the same physician, trained in another centre before starting himself and with technical assistance provided by Curon Medical Co for the first 10 procedures in order to obtain a good standard knowledge of the device and control machine.

The lack of 24 hours pH monitoring as a secondary endpoint in this study is explained by the evidence in the short-term follow-up reports that acidic exposure is not constantly influenced by the RF delivery to the LES [9, 15, 16, 21]. Neurolysis with impairment of visceral sensory pathways has been extensively debated in the mechanism of action of RF delivery and may play a role as well as oesophageal exposure to hydrogen ions or the compliance of the gastro-oesophageal junction with increased resistance of LES to a distending pressure [17, 20]. Literature data concerning the long-term followup seem to exclude that oesophageal desensitization could be harmful in the natural history of the RF-treated patient. 

In the study of Reymunde and Santiago, the RF procedure performed in 83 consecutive patients with persistent GERD symptoms, despite medical therapy, resulted in significant improvement of QOL and symptom scores; daily medication usage was reduced from 100% at baseline to 13.6% at 48 months. Moreover, Noar and Lofti-Emran reported on 109 consecutive patients who have reached a complete 4-year followup and in which heartburn scores and GERD health-related quality-of-life questionnaire significantly improved with a high patient satisfaction. Medication use decreased significantly from 100% of patients on twice-daily PPI therapy at baseline to 75% of patients showing elimination of medications or only as-needed use of antacids/over-the-counter PPIs at 48 months. The multicenter Stretta registry study of 558 patients reported significant GERD symptom control and procedure satisfaction superior to that achieved with drug therapy. Subgroup analysis found that this superior effect on symptom control and drug use persisted more than 1 year and that most patients were off all antisecretive drugs at followup. 

Moreover, the profile of patients who are candidates for endoscopic therapy has been elucidated [25]. The estimated 20% of patients who have persistent and inadequately controlled heartburn and regurgitation despite escalating doses of PPI make up the largest group of patients treated with the Stretta procedure thus far. Other patients who are potential candidates for the procedure include the estimated 2% of patients with GERD who cannot tolerate PPI, those patients with persistent or recurrent GERD symptoms after antireflux surgery, and those patients who want a minimally invasive alternative to drug treatment. 

The Stretta procedure is easily feasible, as confirmed by the studies reporting a second procedure in patients previously treated [22, 26] and possibly might be also indicated for recurrent reflux following antireflux surgery, especially in patients who also fail medical management [27]. Although a recent cost-effectiveness analysis stated that an approach of prescribing PPIs appears to be the preferable strategy for GERD [28], the reported long-term clinical results, with improved responder and durability rates, might suggest potentially economic advantages for the Stretta procedure versus the costs of surgery greater than previously estimated.

At this time, in the long-term follow-up studies, the Stretta procedure has been proven to be very safe for the treatment of GERD. Randomized sham-controlled studies as well as single- and multicenter prospective trials have been conducted. The complication rate is 1.5%, less than the published complication rate for laparoscopic fundoplication. Severe but transient gastric paresis has been yet described in the literature [8, 15] as a rare complication of the Stretta procedure, probably due to ablation of vagal terminal fibres in the gastric fundus during RF energy delivery. 

In conclusion, according to literature data, the Stretta procedure is the most widely documented and implemented endoscopic procedure for GERD and our study also confirms that RF energy delivery is safe and effective, produces durable and significant improvement in GERD symptoms and quality of life, as well as reduces the use of antireflux medication, with negligible morbidity.

##  Conflict of Interests

This is to certify that Luca Dughera, MD, Navino Monica, MD, Cassolino Paola, MD, De Cento Mariella, MD, Cacciotella Luca, MD, Chiaverina Michele, MD, Cisarò Fabio, MD, have no conflict of interests or financial ties to disclose.

## Figures and Tables

**Table 1:** Baseline characteristics of treated patients (*n* = 56)^a^.

Mean age (yr) 42 ± 14
Male sex, *n* (%) 36 (64.2)
Mean heartburn score 3.8 ± 1.9
Mean HRQL score^b^ 29 ± 9
Mean SF-36 mental 40 ± 7
Mean SF-36 physical^c^ 41 ± 7
Daily PPI use, *n* (%) 56 (100)
Median 24-h pH^d^ 15.85 (5.9–18.2)
Median LES pressure (mmHg) 8.44 (7.2–11.7)
<2 cm hiatal hernia, *n* (%) 28 (50)
Erosive esophagitis at EGD, *n* (%)^e^ 14 (25)

EGD: esophagogastroduodenoscopy; LES: lower esophageal sphincter.

^a^Means report ±1 SD, medians report 25th and 75th percentile ranges, and proportions report absolute numbers (percentiles).

^b^Heartburn and heartburn-related quality of life scores (higher scores for worse symptoms).

^c^SF-36 physical score (higher scores for better function; U.S. general ‘‘healthy group” population mean = 55.3).

^d^Percentage of time esophageal pH was <4 (off antisecretory medications).

^e^Ten patients with grade A and four patients with grade B esophagitis according to the Los Angeles classification.

**Table 2:** Negative outcomes of the STRETTA procedure.

	*n* (total *n* = 56)	%
Chest pain	15	26.7
Mild fever (<38°C)	4	7.1
Transient nausea/vomiting	6	10.7
Transient dysphagia	4	7.1
Prolonged gastroparesis^a^	1	1.7
Perforation	0	—
Mucosal lacerations	0	—
Bleeding requiring transfusion	0	—
Deaths	0	—

^a^In a 52-years-old male patient with IEM and delayed gastric emptying (complete resolution within 8 weeks).
